# Simultaneous development of Guillain–Barre syndrome and bacterial meningitis as complications of pneumonia caused by *Staphylococcus aureus*: a case report

**DOI:** 10.1186/s13256-025-05176-4

**Published:** 2025-05-12

**Authors:** Yuliia Solodovnikova, Anastasiia Revurko, Svitlana Oliinyk, Anatoliy Son

**Affiliations:** Department of Neurology and Neurosurgery, Odesa National Medical University, Odesa, Ukraine

**Keywords:** Guillain–Barre syndrome, Bacterial meningitis, Staphylococcus aureus pneumonia, Case report

## Abstract

**Background:**

Guillain–Barre syndrome is an acquired inflammatory polyradiculoneuropathy that often follows gastrointestinal infection. A review of available literature revealed only few cases where Guillain–Barre syndrome developed after different types of meningitis. Furthermore, there are isolated cases of Guillain–Barre syndrome combined with meningeal irritation. This is the first reported case of Guillain–Barre syndrome and bacterial meningitis occurring simultaneously as complications of community-acquired pneumonia.

**Case presentation:**

We report the case of a 77-year-old Ukrainian male patient who presented to the hospital with clinical symptoms of pneumonia. Over the next day, he developed clinical symptoms of acute symmetric ascending flaccid tetraparesis. A few days later, synkinesis-like movements appeared in the paralyzed lower limbs. Microbiological studies of the cerebrospinal fluid identified antibiotic-sensitive *Staphylococcus aureus*. The patient received treatment with intravenous immunoglobulin and combination antibacterial therapy. The patient was discharged with improvement.

**Conclusion:**

In this case, a potentially life-threatening condition, such as bacterial meningitis, was asymptomatic and clinically unrecognized. It is important to recognize atypical cases of Guillain–Barre syndrome to achieve early diagnosis and treatment.

## Background

Guillain–Barre syndrome (GBS) is an immune-mediated polyradiculoneuropathy that typically develops several weeks after an infectious disease. GBS is characterized by acute flaccid ascending paralysis [1]. Globally, GBS affects approximately 100,000 people per year [2]. It most commonly occurs after infection caused by *Campylobacter jejuni* [1]. A review of available literature revealed rare cases where GBS developed after leptospirosis, bacterial meningitis, Zika virus-associated aseptic meningitis, tuberculous meningitis, and meningococcal meningitis [3–7]. Moreover, we have found isolated case reports of the combination of GBS with meningeal irritation without typical changes in the cerebrospinal fluid (CSF), which is specific to bacterial meningitis [8–10].

In the present clinical case, we describe the first case in available literature of the simultaneous development of GBS and bacterial meningitis as complications of pneumonia caused by *Staphylococcus aureus*.

## Case presentation

A 77-year-old Ukrainian male patient presented to the hospital with extreme generalized weakness, chest pain, a nonproductive cough, a temperature of 37.2 °C, shortness of breath, and urinary retention, all of which had developed over the past 3 weeks. The patient has comorbidities, including diabetes mellitus and atrial fibrillation. The patient had been taking nonsteroidal antiinflammatory drugs but had not received antibiotic therapy. A computed tomography (CT) scan revealed right-sided polysegmental pneumonia with pleural effusion. The laboratory findings were typical for bacterial infection and revealed neutrophilic leukocytosis, mild anemia, and elevated C-reactive protein and procalcitonin levels (Table 1).

**Table 1:** Results of the patient’s laboratory tests at admission and discharge

Tests	Result		Units
	Upon admission	Upon discharge	
WBC	18.8	10.8	10^9^/L
RBC	3.24	3.43	10^12^/L
Hemoglobin	102	95	g/L
Platelets	280	193	10^9^/L
Band neutrophils	16	9	%
Segmented neutrophils	71	72	%
Eosinophils	0	0	%
Basophils	0	0	%
Monocytes	3	4	%
Lymphocytes	10	15	%
ESR	28	55	mm/h
C-reactive protein	238.8	97.6	mg/L
Procalcitonin	0.7	0.2	ng/mL

WBC - white blood cells, RBC - red blood cells, ESR - erythrocyte sedimentation rate

Upon admission, the patient was diagnosed with polysegmental community-acquired pneumonia. The results of the initial neurological examination were unremarkable (Table 2).

**Table 2:** Neurological examination of the patient

Characteristic		Result				
		Upon admission	On day 2 of hospitalization	On day 3 of hospitalization	On day 6 of hospitalization	Upon discharge
Consciousness		Alert	Alert	Alert	Alert	Alert
Cognitive function		Intact	Intact	Intact	Intact	Intact
Upper motor neuron		Intact	Intact	Intact	Babinski signs	Intact
Lower motor neuron	Bulbar muscles	Intact	Intact	Moderate dysarthria, dysphonia, dysphagia	Intact	Intact
	Muscle strength of axial and neck muscles	5/5	5/5	2/5	4/5	5/5
	Muscle strength in the upper extremities	5/5	2/5	2/5	3/5	4/5
	Muscle strength in the lower extremities	5/5	0/5	0/5	1/5	2/5
Autonomic dysfunction		Urinary retention	Urinary retention	Urinary retention	Urinary retention	Intact
Sensitivity		Intact	A “stocking-glove” pattern of sensory loss	A “stocking-glove” pattern of sensory loss	A “stocking-glove” pattern of sensory loss	Intact
Meningeal signs		Not elicited	Not elicited	Synkinesis-like movements	Synkinesis-like movements, nuchal rigidity, positive Kernig’s and Brudzinski’s signs	Not elicited

The patient’s treatment was initiated with cefepime 1.0 g twice daily. Over the following day, he developed clinical symptoms of acute symmetric ascending flaccid tetraparesis (graded 2/5 in the upper extremities and 0/5 in the lower extremities), bilateral loss of deep tendon reflexes, and a “stocking–glove” pattern of sensory loss. Autonomic dysfunction (urinary retention) persisted. At the same time, the patient’s level of consciousness was intact, cognitive functions were preserved, and meningeal signs were not elicited. Furthermore, there were no signs of upper motor neuron involvement (Table 2). GBS was suspected. The nerve conduction study was not performed because of technical issues. The lumbar puncture was performed to confirm GBS. The cerebrospinal fluid (CSF) was purulent in appearance, turbid, milky-yellow in color, and had a low opening pressure due to the extremely increased viscosity. The laboratory test of the CSF is described below. Microbiological studies of the CSF identified antibiotic-sensitive *S. aureus* (Table 3).

**Table 3:** Results of the cerebrospinal fluid analysis

Tests	Results				Units
	On day 2 of hospitalization	On day 3 of hospitalization	On day 6 of hospitalization	On day 20 of hospitalization	
Color	Milky-yellow	Milky-yellow	Milky-gray	Clear	
Clarity	Turbid	Turbid	Turbid	Clear	
Proteins	1.65	2.7	6.6	0.66	g/L
Nonne-Apelt reaction	+	+ + +	+ + + +	+	
Pandy test	+	+ + + +	+ + + +	+	
Chloride	106.7	103		113.8	mmol/L
Glucose	0.5	1.3		0.71	mmol/L
WBC count	93	1585	1707	6	(per µL)
Neutrophils	80%	92%	84%	65%	%
Lymphocytes	20%	8%	16%	35%	%
RBC count	101	Nil	12-18-25	0-2-4	(per µL)
Microbial culture	*S. aureus*	*S. aureus*	*S. aureus*	Negative	

WBC count - white blood cells count, RBC count - red blood cells count

On day 3, motor disorders progressed to bulbar palsy and peripheral paresis of the axial trunk and neck muscles (dropped head syndrome), along with the fulminant onset of diffuse amyotrophies, without any worsening of other neurological deficits. In addition, during active or passive movements of the upper limbs, involuntary movements resembling synkinesis were observed in the paralyzed lower limbs (Table 2). The patient was treated with intravenous immunoglobulin at 0.4 g/kg/day for 5 days. The patient also received combination antibacterial therapy with cefepime (2.0 g twice daily) and linezolid (600 mg twice daily) administered intravenously, which was subsequently replaced by meropenem (2.0 g three times daily) and levofloxacin (500 mg twice daily). The patient’s condition gradually improved. Over the next few days, muscle strength increased in a descending pattern. Concurrently, stiffness of the occipital muscles and bilateral Babinski signs appeared simultaneously with the ascending emergence of meningeal signs, progressing from nuchal rigidity during the regression of axial muscle paralysis to positive Kernig’s and Brudzinski’s signs as movement returned to the legs. Involuntary movements resembling synkinesis in the lower limbs persisted until the complete resolution of nuchal rigidity (Table 2). The patient was discharged with clinical and radiological resolution of pneumonia, positive laboratory trends, sterile CSF, and partial recovery of motor deficits: muscle strength in the upper extremities was graded 4/5, while in the lower extremities, it was graded 2/5 (Tables 1, 2). Thus, the patient concurrently developed GBS and asymptomatic bacterial meningitis. The flaccid tetraparesis precluded the manifestations of meningeal syndrome.

## Discussion and conclusion

This is the first reported case of GBS and bacterial meningitis occurring simultaneously as complications of community-acquired pneumonia. Community-acquired pneumonia caused by *S. aureus* represents 1–2% of all such pneumonias. It is associated with a high mortality rate, especially in patients with comorbidities. There is only one other documented case in literature describing an association between *S. aureus* infection and GBS in an adolescent patient with community-acquired necrotizing pneumonia [11]. The pathogenesis of GBS is based on molecular mimicry, the production of ganglioside antibodies, and damage to peripheral nerve targets [12]. The pathogenesis of meningitis involves the penetration of *S. aureus* through the blood–brain barrier due to hematogenous dissemination. It should be noted that diabetes is associated with an increased level of *S. aureus* colonization and is a predictor of poor outcomes in patients with meningitis. Hyperglycemia causes cerebral edema and contributes to the blood‒brain barrier disruption [13].

Our patient had all the following clinical manifestations of GBS: acute onset, the presence of a progressive monophasic illness for up to 4 weeks, a history of respiratory tract infection, symmetrical weakness in the upper and lower limbs, areflexia, paresthesia/numbness, cranial neuropathies (bulbar muscles), and autonomic dysfunction [14]. The severity of the clinical manifestations of GBS concealed the typical clinical signs of bacterial meningitis at disease onset.

The appearance of synkinesis-like movements should be interpreted as a possible new meningeal sign. Regression of the manifestations of acute symmetric ascending flaccid tetraparesis correlated with the appearance of meningeal syndrome manifestations.

In this case, a potentially life-threatening condition, such as bacterial meningitis, was initially asymptomatic and clinically unrecognized. Therefore, the atypical presentation of the simultaneous development of GBS and bacterial meningitis as complications of *S. aureus*-associated pneumonia should be kept in mind while formulating the diagnosis and management plan for these patients.

## Data Availability

Data sharing is not applicable to this article, as no datasets were generated or analyzed during the current study.

## Abbreviations

GBS: Guillain–Barre syndrome
CSF: Cerebrospinal fluid
CT: Computed tomography
WBC: White blood cells
RBC: Red blood cells
ESR: Erythrocyte sedimentation rate
